# Supplementary material for: Dynamic transcriptomic profiles of zebrafish gills in response to zinc supplementation
Source: BMC Genomics. 2010 Oct 11;11:553. doi: 10.1186/1471-2164-11-553 (PMC3091702; doi:10.1186/1471-2164-11-553)
Supplement: Additional file 2 — Interactive Direct Interaction Network representing the molecular interactions between zinc, copper, iron, calcium and proteins encoded by transcripts changed by zinc supplementation. Mini web-site containing index.html and hyperlinked pages in subdirectory describing a Direct Interaction Network automatically generated based on curated interactions contained within the proprietary PathwayArchitect database. Ovals represent proteins and the circles symbolize metal ions. Objects are coloured by their abundance in zebrafish at the time-point they were significantly different from the control is a scale from -4 fold (dark green) to +4 fold (dark red). Where significant differences were found at more than one time-point, the colour overlay shows expression at the first instance. Dark blue squares denote 'binding', and light blue squares 'expression'; green squares stand for 'regulation', green diamonds for 'metabolism', and green circles for 'promoter binding'. Arrow heads indicate directionality of the interaction where annotated. All nodes and edges can be further interrogated by selecting the relative area of the image. [file 1471-2164-11-553-S2.zip › PathwayArchitect Zn xs DIN/409465.html]

# REGULATION:

|  |  |
| --- | --- |
| Type | REGULATION |
| Effect | Negative |


---

|  |  |
| --- | --- |
| Score | 0 |


---

|  |  |
| --- | --- |
| Reference Count | 6 |


---

|  |  |
| --- | --- |
| Mechanism | Unknown |


---

|  |  |
| --- | --- |
| Reference:0 || Sentence | "The results suggest that Ca2+ transport by the Na+/Ca2+ exchanger is inhibited by concentrations of zinc thought to be attained extracellularly after excitation." |
| PMID | 9804322 |
| Year | 1998 |
| Species | Rat |
| Journal | Neuroreport |
| RefScore | 0 |
| Source | PArchNLP |
  |
|  |  |


---

|  |  |
| --- | --- |
 Reference:1 || Sentence | "Probably, salicylate and Zn deficiency inhibit Ca2+ mobilization by parathyroid hormone in bone." |
| PMID | 2484541 |
| Year | 1988 |
| Species | Rat |
| Journal | Biol Trace Elem Res |
| RefScore | 0 |
| Source | PArchNLP |
  ||  |  |


---

|  |  |
| --- | --- |
 Reference:2 || Sentence | "Finally zinc competitively inhibits the calcium-dependent release of transmitter by inhibiting the entry of Ca2+ into the nerve terminals." |
| PMID | 2192611 |
| Year | 1990 |
| Journal | Ann N Y Acad Sci |
| RefScore | 1 |
| Source | PArchNLP |
  ||  |  |


---

|  |  |
| --- | --- |
 Reference:3 || Sentence | "Zinc binding to calgranulin C induces a remarkable increase in the protein affinity for calcium; in the absence of zinc, the protein binds 1 Ca2+/monomer with a binding constant of about 2 x 10(4) M-1, whereas the Zn(2+)-loaded form binds 2 Ca2+/monomer with Ka values of approximately 3 x 10(7) and 6 x 10(4) M-1." |
| PMID | 7961855 |
| Year | 1994 |
| Species | Human |
| Journal | J Biol Chem |
| RefScore | 1 |
| Source | PArchNLP |
  ||  |  |


---

|  |  |
| --- | --- |
 Reference:4 || Sentence | "Zinc probably inhibits the entry of Ca2+ into the nerve terminals, thereby inhibiting transmitter release." |
| PMID | 2897107 |
| Year | 1987 |
| Species | Mouse |
| Journal | Pflugers Arch |
| RefScore | 0 |
| Source | PArchNLP |
  ||  |  |


---

|  |  |
| --- | --- |
 Reference:5 || Sentence | "Thus physiological concentrations of zinc inhibit the release of histamine from human basophils and lung mast cells, presumably by blocking Ca2+ uptake induced by anti-IgE activation." |
| PMID | 2425567 |
| Year | 1986 |
| Species | Human |
| Journal | Agents Actions |
| RefScore | 1 |
| Source | PArchNLP |
  |


---

|  |  |
| --- | --- |
